# Self-Reactivities to the Non-Erythroid Alpha Spectrin Correlate with Cerebral Malaria in Gabonese Children

**DOI:** 10.1371/journal.pone.0000389

**Published:** 2007-04-25

**Authors:** Vincent Guiyedi, Youri Chanseaud, Constantin Fesel, Georges Snounou, Jean-Claude Rousselle, Pharat Lim, Jean Koko, Abdelkader Namane, Pierre-André Cazenave, Maryvonne Kombila, Sylviane Pied

**Affiliations:** 1 Unité d'Immunophysiopathologie Infectieuse, URA CNRS 1961, Université Pierre et Marie Curie Paris, Institut Pasteur, Paris, France; 2 Département de Parasitologie-Mycologie-Médecine Tropicale, Faculté de Médecine, Université des Sciences de la Santé, Libreville, Gabon; 3 Instituto Gulbenkian de Ciência, Oeiras, Portugal; 4 Parasitologie comparée et Modèles expérimentaux, Département Ecologie et Gestion de la Biodiversité, Muséum National d'Histoire Naturelle, Paris, France; 5 Plate-Forme de Protéomique, Pasteur Génopole, Institut Pasteur, Paris, France; 6 Hôpital Pédiatrique d'Owendo, Libreville, Gabon; Federal University of São Paulo, Brazil

## Abstract

**Background:**

Hypergammaglobulinemia and polyclonal B-cell activation commonly occur in *Plasmodium sp.* infections. Some of the antibodies produced recognize self-components and are correlated with disease severity in *P. falciparum* malaria. However, it is not known whether some self-reactive antibodies produced during *P. falciparum* infection contribute to the events leading to cerebral malaria (CM). We show here a correlation between self-antibody responses to a human brain protein and high levels of circulating TNF alpha (TNFα), with the manifestation of CM in Gabonese children.

**Methodology:**

To study the role of self-reactive antibodies associated to the development of *P. falciparum* cerebral malaria, we used a combination of quantitative immunoblotting and multivariate analysis to analyse correlation between the reactivity of circulating IgG with a human brain protein extract and TNFα concentrations in cohorts of uninfected controls (UI) and *P. falciparum*-infected Gabonese children developing uncomplicated malaria (UM), severe non-cerebral malaria (SNCM), or CM.

**Results/Conclusion:**

The repertoire of brain antigens recognized by plasma IgGs was more diverse in infected than in UI individuals. Anti-brain reactivity was significantly higher in the CM group than in the UM and SNCM groups. IgG self-reactivity to brain antigens was also correlated with plasma IgG levels and age. We found that 90% of CM patients displayed reactivity to a high-molecular mass band containing the spectrin non-erythroid alpha chain. Reactivity with this band was correlated with high TNFα concentrations in CM patients. These results strongly suggest that an antibody response to brain antigens induced by *P. falciparum* infection may be associated with pathogenic mechanisms in patients developing CM.

## Introduction

Malaria remains one of the leading causes of death in most of sub-Saharan Africa, and mortality rates are particularly high for children under the age of five years, pregnant women and non-immune individuals [1]. Fatal outcome occurs nearly exclusively in patients infected with *Plasmodium falciparum* who progress to severe malaria [1], [2]. Severe anemia and cerebral malaria (CM) are the most prevalent types of severe *P. falciparum* malaria, and CM displays the more acute clinical spectrum. In CM, the sequestration of mature *P. falciparum*-infected red blood cells to the cerebral endothelium is clearly a central event in the pathogenesis. However, the sequences of pathological events leading to fatal malaria in humans remain poorly characterized. Gaining an understanding of the mechanisms involved in CM will be crucial to the development of new preventive or curative therapies and/or vaccines, as well as to defining prognostic markers.

It has been recently shown that production of autoantibodies to brain voltage-gated calcium channels, but not other calcium channels, increased with severity of *P. falciparum* infection in Kenyan children [3]. Antibodies to central nervous system proteins have been associated with seizure and epilepsies in several autoimmune diseases and are thought to play a role in the pathology [4]. Hypergammaglobulinemia and polyclonal B-cell activation commonly occur in *Plasmodium sp.* Infections [5]. Some of the antibodies produced recognize self-components from various tissues and organs, such as erythrocytes, lymphocytes, nucleic acid structures, cytoskeleton, smooth muscle, heart and thyroid [6], [7]–[10]. Antibodies against phospholipid (PL), cardiolipin (CL), ssDNA, dsDNA, and rheumatoid factor are correlated with disease severity in *P. falciparum*-infected patients [6], [9], [11]–[13], and Coombs' anti-globulin test is positive in *P. falciparum*-infected individuals suffering from severe anemia [14], [15]. It is unclear whether self-reactive antibodies play a role in protective immunity against blood-stage parasites [16]. Moreover, it is not known whether the presence of these autoantibodies is a consequence of the infection or whether it contributes to the events leading to severe and particularly to *P. falciparum* cerebral malaria.

Another factor thought to influence clinical outcome of malaria is the fine balance between the pro- and anti-inflammatory cytokines, such as TNFα, IFNγ and IL-10 produced during the infection, that modulate parasite–induced immune responses [17], [18]. In particular, tumor necrosis factor α (TNFα) is thought to play an important role in CM physiopathology by inducing changes in cerebral endothelial cells leading to the surface expression of adhesion molecules thereby enhancing parasitized erythrocyte sequestration [17], [18]. The adhesion of parasitized red blood cells to the brain vascular endothelium is considered to lead to a decrease in cerebral blood flow and to contribute to the induction of brain damage and coma [17], [19]–[22]. Anti-inflammatory cytokines, such as IL-10, contribute also to the regulation of the inflammatory response during malaria [18]. Thus, the outcome of *P. falciparum* infection may depend on a fine balance between appropriate and inappropriate induction of theses immune regulatory factors.

Whether IFNγ, TNFα and IL-10 regulate the self-reactive antibody response during malaria, as is the case for primary biliary cirrhosis, remains to be shown [23].

We wished to investigate a) whether autoantibodies directed against brain tissues occur in *P. falciparum* malaria, and if so to identify the host molecules recognised, b) if there is a relationship between autoantibody levels and clinical symptoms, and c) whether self-reactive antibodies, in association with a defined pattern of circulating cytokines, are associated to the development of CM. We therefore recruited three groups of patients developing uncomplicated (UM), severe non-cerebral (SNCM) or cerebral malaria attending the Owendo Pediatric Hospital and Libreville Hospital Center in Gabon. We further investigated whether levels of circulating IFNγ, TNFα and IL-10 in these groups of patients were associated with the level of IgG self-reactivitive antibodies response.

## Methods

### Patients and Methods

#### Study population

Patients were included in the study only after informed consent had been obtained from their parents, after admission at the Owendo Pediatric Hospital (OPH) and Libreville Hospital Center (LHC) in Gabon, between 1996 and 1999. The ethics committee of the Gabon Health Office approved this study. Patients were assigned to the various groups on the basis of World Health Organization guidelines for the definition of uncomplicated and severe malaria. The children included in this study were aged between 2 months and 5 years, and fell into three groups for *P. falciparum* malaria: 1) uncomplicated malaria (UM), 2) severe non-cerebral malaria (SNCM) with severe anemia (hemoglobin level < 5 g/dl) or hypoglycemia (glycemia<2.2 mmol/ml), and 3) cerebral malaria (CM) with a Blantyre Coma Score inferior or equal to 2, or three convulsive episodes during the 24 hours before admission, with a post-critical comatose stated lasting>15 minutes. We also studied two control groups—the uninfected group (UI)—comprising healthy children with *P. falciparum*-negative thin blood smears, and European uninfected group (EUIC), who had never been exposed to malaria. All patients presenting diseases other than malaria were excluded from the study. All the UI subjects (18) were from the same area of Libreville City, and the patients in the UM (57), SNCM (24) and CM (14) groups were all recruited from the OPH and LHC hospitals. During the recruitment period, 7% (8/113) of the patients died in CM group. There was no significant difference in mean age and in sex ratio between the children in the various groups.

Patients were treated with amodiaquine or quinine, depending on disease severity. Children with uncomplicated malaria were given oral amodiaquine (25 mg/kg) for three days; those with severe malaria received intravenous quinine (25 mg/kg/day) for five days. Children with severe anemia underwent blood transfusion.

#### Blood sample collection and parasite assessment

Venous blood from each patient was collected into EDTA in sterile vacutainers on the day of hospitalization (day-0, before any treatment), at day-7 and day-30. Plasma was obtained by centrifuging blood samples at 5000 g for 15 min, and stored at −80°C until use. Parasitemia was assessed by counting asexual forms of *P. falciparum* on thin blood smears under a light microscope after staining with 10% Giemsa solution. Parasitemia is expressed as the percentage of infected red blood cells.

#### Quantification of total plasma IgG and cytokines

Plasma total IgG of the patients were quantified by “Sandwich type ELISA” method as described in detail elsewhere [24], [25]. A sandwich-type ELISA was used to determine IFNγ, TNFα and IL-10 levels, using a kit (OptEIA set, Pharmingen, BD Bioscience, France), according to the manufacturer's recommendations.

#### Brain extract preparation, gel electrophoresis and immunoblotting

Patterns of recognition of brain proteins by plasma IgG were detected by quantitative immunoblotting (PANAMA-blot method), using a protein extract from the brain of a healthy individual as the source of antigens as previously described [26], [27]. Briefly, normal human brain protein extracts (about 300 µg protein/gel) were separated by a standard SDS-PAGE in a 10% polyacrylamide gel. The proteins were transferred onto nitrocellulose membranes (Schleicher&Schuell, Dassel, Germany) by semi-dry electrotransfer (Pasteur Institute, Paris, France). Membranes were then incubated with patient plasma samples diluted 1:20 in PBST (non-adjusted assay), or to a total IgG concentration of 200 µg/ml (adjusted assay), in a Cassette Miniblot System (Immunetics, Cambridge, MA, USA). The immunoglobulin reactivities were detected by incubation with chain-specific secondary rabbit anti-human IgG coupled to alkaline phosphatase (Sigma-Aldrich, Saint Quentin Fallavier, France). Dried membranes were scanned with a high resolution scanner (600 DPI). We used a mixture of diverse Gabonese plasma preparations diluted 1/20 as the standard plasma sample.

#### Protein identification by mass spectrometry

Protein bands of interest were localised following migration on 10% acrylamide gels and staining with Coomassie blue. The proteins present in these bands were identified by peptide mass fingerprinting with a matrix-assisted laser desorption ionization-time of flight (MALDI-TOF) mass spectrometer (MS), Voyager DE-STR (Applied Biosystems, Framingham, MA, USA) [28].

#### Statistical analysis

Immunoblot data were analyzed by multivariate statistical methods, using IGOR software (Wavemetrics, Lake Oswego, OR), including specially written software packages. The standard migration scale was divided into sections around individual peaks of immunoreactivity. Section-wise absorbance values were subjected to principal component analysis (PCA), based on covariance calculation. For quantitative comparisons between groups, we used either Mann-Whitney (between two groups) or Kruskal-Wallis tests (>2 groups). Qualitative association was tested by Pearson'sχ^2^ test. The association between continuous quantitative parameters was assessed by linear regression, with the exception of correlations between two different types of parameters such as reactivity and cytokine profiles, which were tested by Spearman's rank correlation. *P* values<0.05 were considered significant.

## Results

### Diversity of IgG reactivity with brain antigens in the various clinical groups

**Figure 1:**
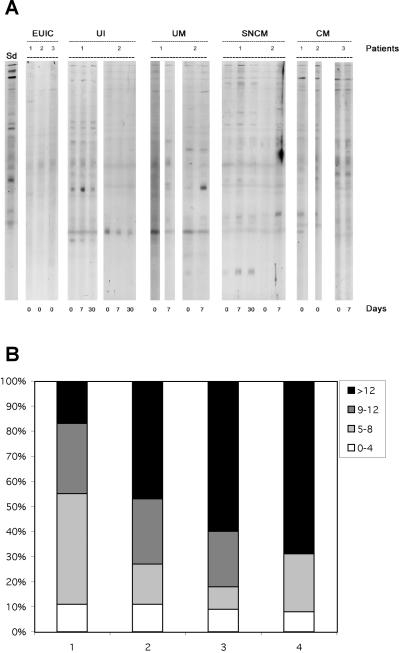
IgG immunoreactivity profiles from malaria patients. A. Example of reactivities of IgG from patients sera in the EUIC (3), UI (2), UM (2), SNCM (2) and CM (3) groups, respectively at day 0, day 7 and/or day 30 for each patient. B. Frequency of patients in each group recognizing ranges of 0–4, 5–8, 9–12 and more than 12 bands, respectively. 1: UI, 2: UM, 3: SNCM, 4: CM.

We carried out an adjusted assay, in which patient plasma was systematically diluted to an identical total IgG concentration (200 µg/ml) before testing, so as to determine the proportion of total IgG accounted for autoantibodies. Figure 1 shows typical examples of the immune profiles obtained with the brain extract, for several patients of each group, on days 0, 7 and 30. Reactivity patterns were more diverse in the UM, SNCM and CM groups than they were in the UI and EUIC (European uninfected controls) group, but these did not change with the time of collection (figure 1a). The median number of cerebral antigens recognized by plasma IgG was significantly higher in UM (p = 0.02), SNCM (p = 0.01) and CM (p = 0.02) than in UI subjects, whereas the difference in the number of bands recognized did not differ significantly between UM, SNCM and CM patients (data not shown). We determined the proportion of patients in each group with plasma IgG recognizing a particular number of bands (figure 1b). The proportion of individuals with a large number of bands (>12) was higher in CM (71%) and SNCM (58%) than in UM (49%) and UI (17%) subjects. The overall difference between groups was significant (χ^2^ = 11.2, p = 0.01). Thus, the number of brain antigens recognized by circulating IgG from *P. falciparum*-infected individuals increased with disease severity, with the sera of most UI subjects reacting with less than eight bands (figure 1b).

### Relationship between the size of the IgG repertoire directed against brain antigens and disease severity

**Figure 2:**
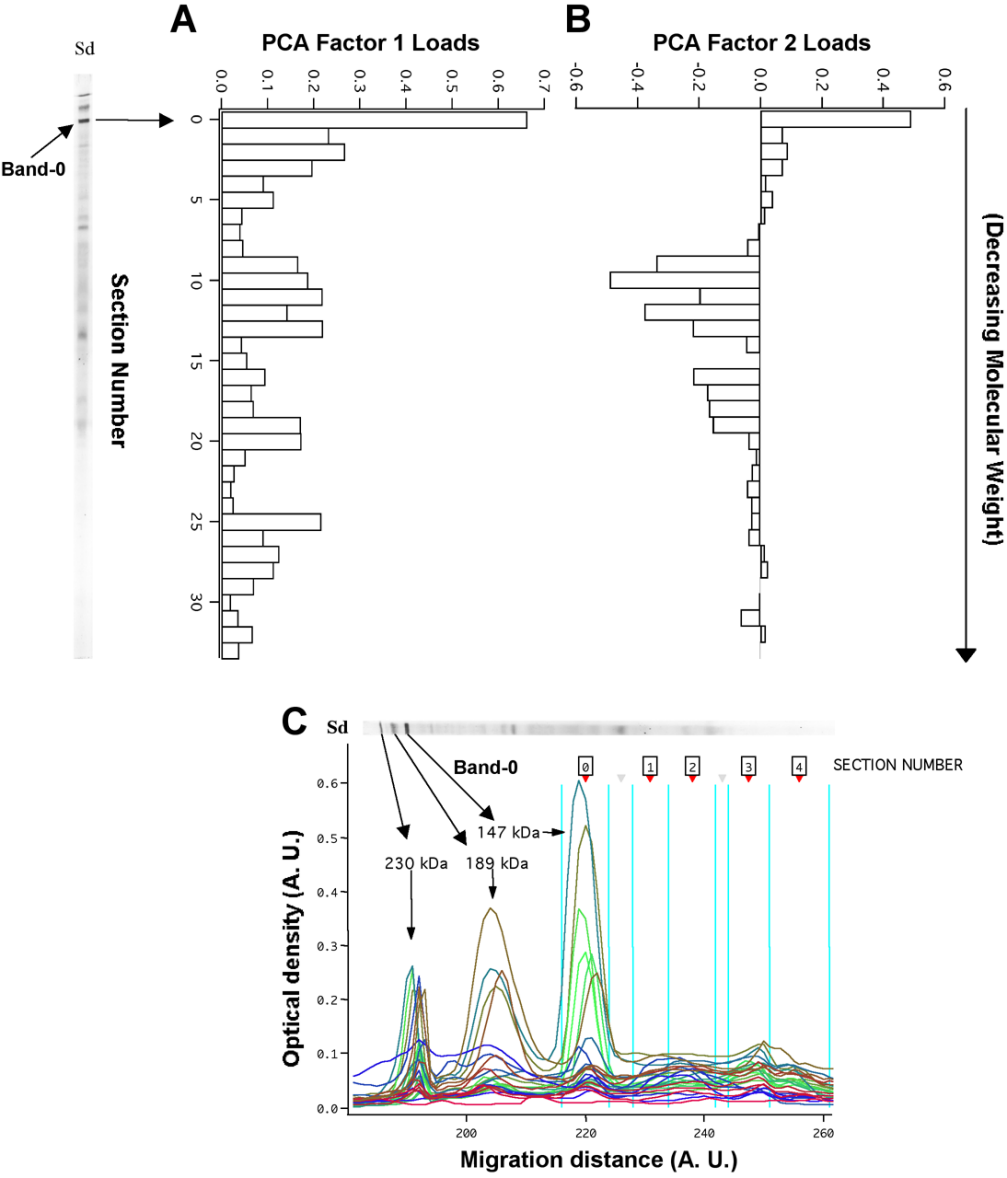
PCA factor loads. Relative contributions of reactivity bands to the first two PCA factors calculated on unadjusted profiles of IgG immunoreactivity to brain proteins separated by 10% SDS-PAGE. A. PCA factor 1. B. PCA factor 2. C. localisation of the band-0 on Western blot profile obtain after the computer analysis of membrane N°5. Bands are ordered from high to low molecular weight (between about 230 kDa and 20 kDa).

**Figure 3:**
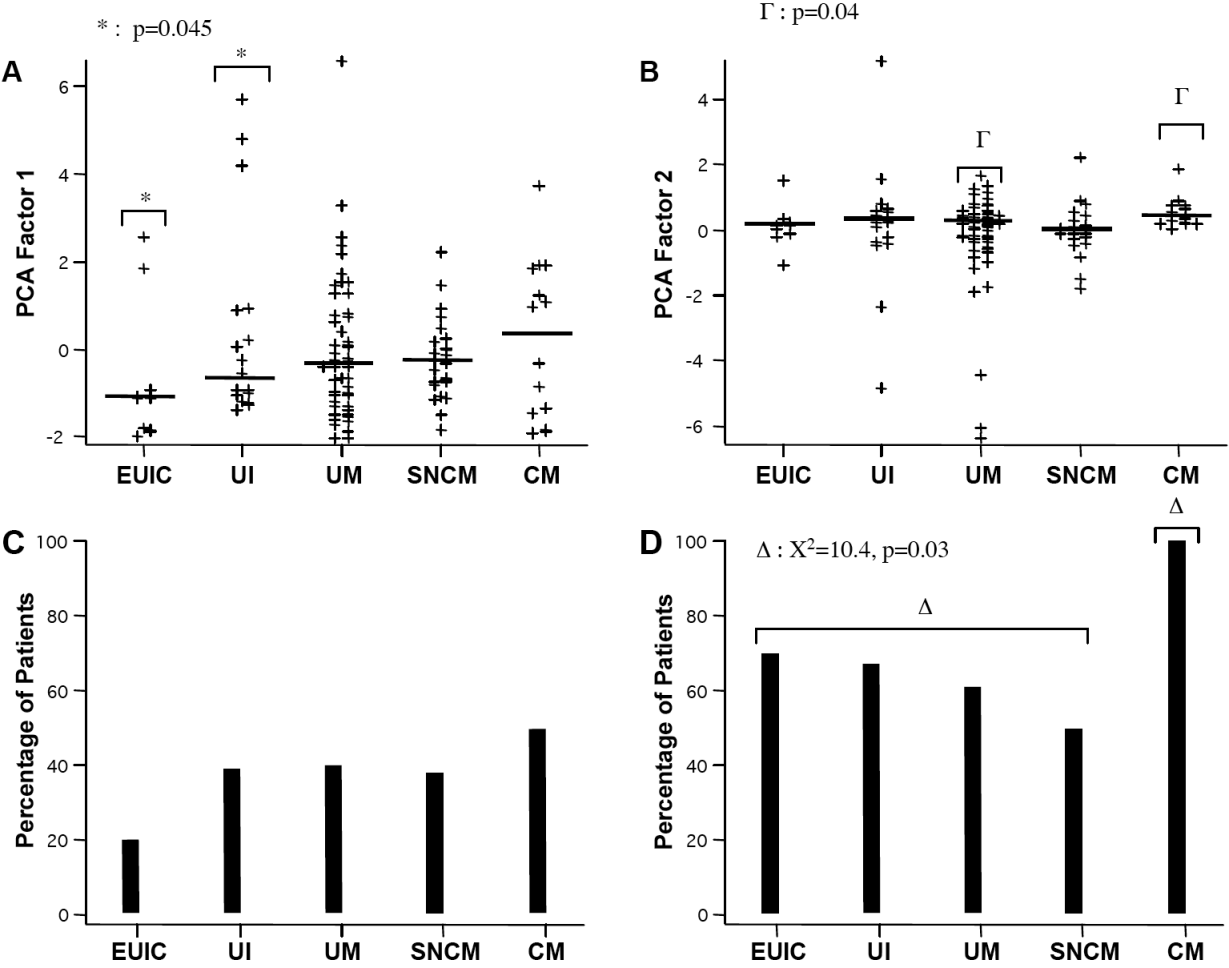
PCA factor scores from unadjusted IgG immunoreactivity profiles. A. Groupwise distribution of factor 1 scores. B. Groupwise distribution of factor 2 scores. C. Frequency of patients in each group with above-average factor 1 scores. D. Frequency of patients in each group with above-average factor 2 scores.

We investigated the distribution of circulating IgG reactivities to brain proteins by carrying out a second quantitative immunoblot assay, using the same method as above but analyzing every plasma sample at a fixed dilution of 1/20 (non–adjusted assay). In this assay, we included 10 European children of similar ages who had never been exposed to malaria. We then analyzed the patterns of reactivity of the patients by principal component analysis (PCA), fitted to the Gabonese data. In PCA, the components are identified in decreasing order of importance. Thus, by definition, the first two components identified account for a large proportion of total reactivity. Factor 1 was characterized by overall positive factor loads (figure 2a). Factor 1 scores, therefore, provide a quantitative measure of total reactivity. Factor 2 scores mostly reflected the recognition of one particular section (the section-0, including the band-0) rather than the others (figure 2b, figure 2c). Factor 1 and factor 2 scores were higher in CM than in UI, UM and SNCM patients (figure 3a and 3b). Factor 1 scores were significantly lower in EUIC than in Gabonese UI (p = 0.045). No significant difference was observed between the UI, UM, SNCM and CM groups (figure 3a). Factor 2 scores differed significantly between CM and SNCM patients (p = 0.001) and between CM and UM patients (p = 0.04) (figure 3b).

The proportion of patients with high factor 1 scores (and therefore high overall anti-brain reactivity) appeared to be highest for CM patients, but no significant difference was found between the groups (figure 3c). The proportion of patients with high factor 2 scores was significantly lower in EUIC, UI, UM and SNCM (70%, 67%, 61%, and 50%, respectively) than in CM (100%) subjects (χ^2^_All Groups_ = 10.4, p = 0.03) (figure 3d). Remarkably, all CM patients had factor 2 scores above the mean.

In addition to the day 0 samples, we also analyzed samples taken from the same individuals (14 UI, 42 UM, 27 SNCM and 3 CM patients) on days 7 and 30 after admission. The factor scores obtained for these samples were determined by projection, using factor loads calculated from day 0 data. Factor 1 scores increased significantly between days 0 and 7 in patients developing malaria whereas, on day 30, reactivity patterns were identical to those on day 7 for most of the children (data not shown). No such increase was observed in factor 2 scores.

### Reactivity to brain proteins according to age, sex, parasitemia and circulating IgG levels

**Figure 4:**
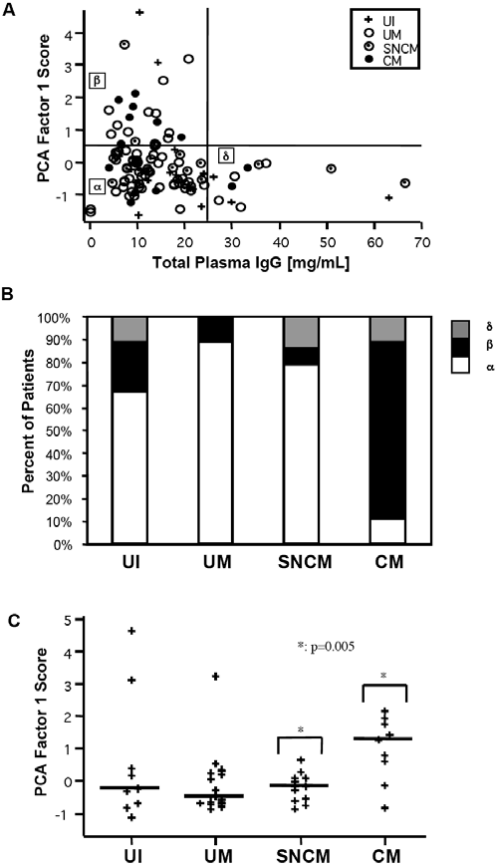
Properties of adjusted IgG anti-brain reactivity in terms of PCA factor 1 scores. A. Relationship to total plasma IgG concentration, and dissection into three subgroups : α (IgG<25 mg/ml; F1<0.5), β (IgG<25 mg/ml; F1>0.5), δ (IgG>25 mg/ml; F1<0.5). B. Frequencies of patients above one year of age in each group, over the three subgroups. C. Groupwise distribution of PCA factor-1 scores among patients older than one year, with horizontal bars indicating median values. The significance of the difference between patients with CM and with non cerebral clinical malaria (UM+SNCM) is indicated.

The reactivity to brain antigens represented by factor 1 scores was significantly correlated with age (R = +0.39, p = 0.001) in the non-adjusted assay, whereas no correlation was found between reactivity profiles, sex and parasitemia (data not shown). However, circulating IgG concentration was found to be significantly correlated with reactivity in all groups (R = +0.21, p = 0.03) except for uninfected controls (data not shown). These correlations with age and total IgG were less significant (p = 0.03 and 0.04, respectively) for PCA calculated from the adjusted assay, previously used for the assessment of diversity, which may therefore better reflect intrinsic repertoire properties. Here, high factor 1 scores were observed only for patients with low total IgG concentrations (Figure 4a). We assigned the children to three subgroups, as follows: α) low anti-brain reactivity and moderate levels of circulating IgG (below 25 mg/ml, corresponding to twice normal IgG concentration in children), β) high anti-brain reactivity and moderate levels of circulating IgG and δ) low anti-brain reactivity and high levels of circulating IgG. Subgroups α and β displayed unequal distributions of UM, SNCM and CM patients. UM and SNCM patients were overrepresented in subgroup α (72% of the UM and 75% of the SNCM patients, but only 36% of the CM patients; χ-squared test for all groups: p = 0.049). Subgroup β included 50% of the CM patients but only 21% of all samples (χ-squared test: p = 0.028), while there was no preferential distribution in subgroup δ.

During the first twelve months of life, the plasma may contain maternal IgG. The differences between the three subgroups were indeed more pronounced when considering only children over the age of one year: subgroup α contained 89% of the UM and 79% of the SNCM patients but only 11% of the CM patients, whereas 78% of CM patients were found in subgroup β (figure 4b), and the association with clinical status was highly significant for both (p = 0.0005 and 0.0004, respectively). CM patients over the age of one year also showed the highest factor 1 scores (Figure 4c), being significantly higher than for SNCM and UM patients (p = 0.005 and 0.009, respectively). These results, where the effect of both age and total IgG concentrations was largely eliminated by the adjusted assay, indicate a specific self-reactive antibody repertoire induced during *P. falciparum* infection.

### IgG reactivity to a high-molecular weight brain antigen is associated with CM

**Figure 5:**
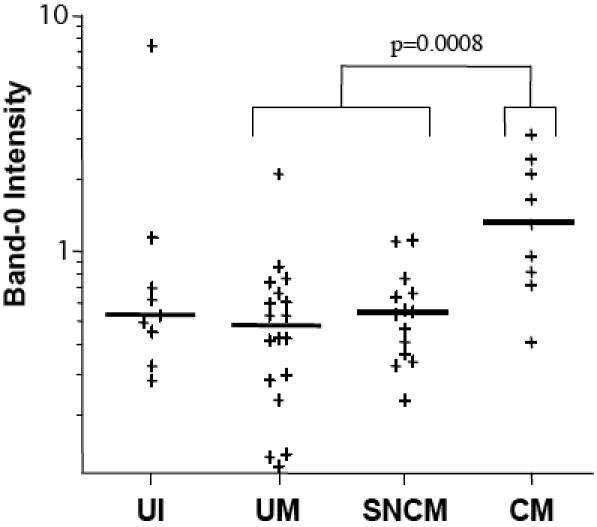
Mass fingerprinting. Proteins from human brain extract were separated on a 10% acrylamide gel. After Coomassie staining, three bands corresponding to reactive bands detected by Western blot were cut and their protein contents analyzed by peptide mass fingerprinting as described in the material and methods section. Three independent experiments were carried out and conducted to identical protein identifications. Results in figure 5 correspond to those obtain in one typical experiment.

**Figure 6:**
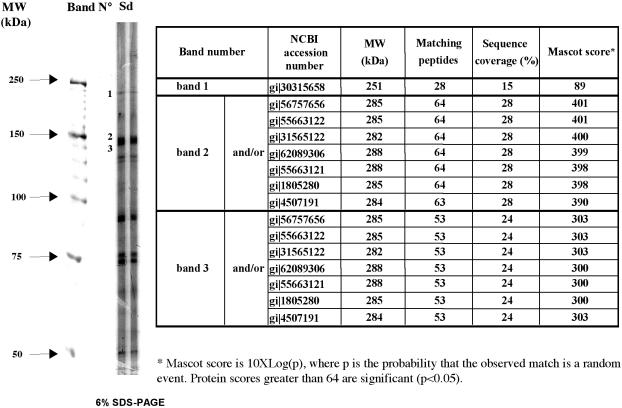
Intensity of IgG reacting to band-0 in children older than one year in each group. Horizontal bars indicate medians; the significant difference between patients with cerebral malaria (CM) and with UM+SNCM is indicated.

Profiles of the reactivity of standard and patient plasma samples with brain proteins were separated into 33 sections on the standardized migration scale. PCA analysis revealed that reactivity with section 0 (band-0), corresponding to a set of high-molecular weight proteins, was the most informative (figure 2). In the two types of assay, this section had the most impact on PCA factor 1. For PCA factor 2, section 0 was distinguished from the rest of the repertoire by a positive load. At least three proteins with estimated molecular weights of 230 kDa, 189 kDa and a double band at 147 kDa on SDS-PAGE were detected in this region in a 6% acrylamide gel with protein size standards (figure 2c, figure 5). In adjusted assays, plasma IgG from children with CM over the age of one year reacted more strongly with band-0 than did plasma IgG from children of the UM and SNCM groups (both p = 0.0008) (figure 6): 90% of the CM patients had a detectable reaction, versus 50% SNCM 44% UI and 39% UM patients (data not shown). The results obtained in unadjusted assays were qualitatively similar, but the differences between CM and UM and between CM and SNCM were less significant (p = 0.02 and 0.01, respectively). Unadjusted reactivity with band-0 proteins increased with age (R = 0.25; p = 0.04) (Data not shown).

### Mass spectrometry identification

Proteins from human brain extract were separated on a 10% acrylamide gel. After Coomassie staining, three bands corresponding to reactive bands detected by Western blot were cut and their protein contents analyzed by peptide mass fingerprinting. In three independent experiments, band 1 corresponded to the beta chain of the non-erythroid spectrin isoform 2 while bands 2 and 3 to seven different isoforms of the alpha chain of the non-erythroid spectrin. In this latter case, it was not possible to distinguish if only one, several or all these isoforms were present in bands 2 and 3 (Figure 5).

### Relationships between IgG reactivity to brain proteins, plasma cytokines concentrations and clinical manifestations of malaria

Plasma IL-10 concentrations were significantly lower in the UI group than in the UM (p = 0.002) and severe malaria groups (p<0.0001), but were similar in the UM, SNCM and CM groups (data not shown). IFNγ concentrations were highest in the UM and CM groups (data not shown). The differences between the CM and UI groups were statistically significant (p = 0.007), whereas no significant differences were observed between the UM, SNCM and CM groups. Plasma TNFα concentrations were significantly higher in the SNCM and CM groups than in the UM group (p = 0.0001 and p = 0.001, respectively). In addition, 75% of CM patients who died had a very high plasma level of TNFα (62.5% from 500 to 1520 pg/ml, and 12.5% from 100 to 500 pg/ml) (data not shown).

**Figure 7:**
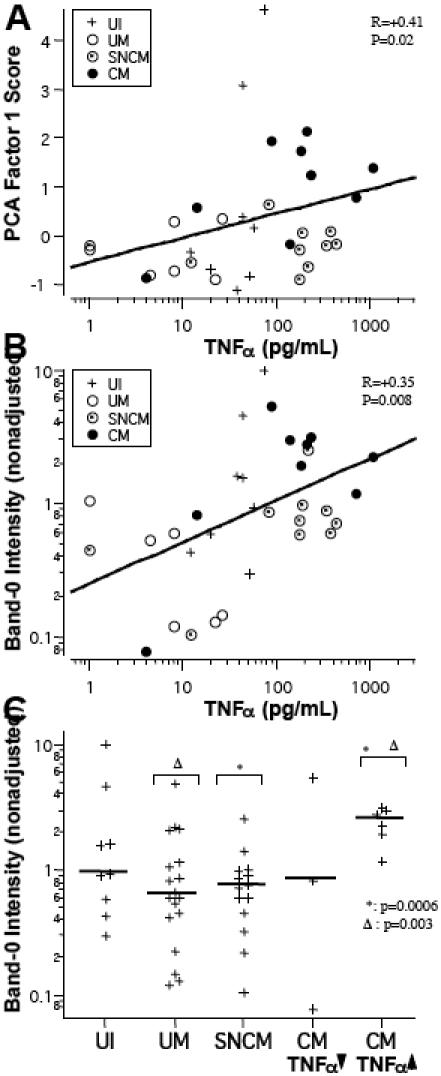
Total reactivity to brain proteins and intensity of IgG reacting to band-0 as a function of plasma TNFα levels in children older than 1 year. A. Positive correlation between PCA factor 1 and TNFα concentrations in the CM group, indicated by dashed regression line. B. Positive correlation between unadjusted reactivity to band 0 and TNFα levels. C. High band-0 reactivity was observed most frequently in CM patients with TNFα levels above 100 pg/mL (indicated as high-TNFα in a separate group on the right). Horizontal bars indicate medians. Only children over the age of 1 year are shown.

We assessed whether IgG autoantibody response and the type of disease were associated with cytokines concentrations in *P. falciparum*-infected patients. Plasma IFNγ and IL-10 concentrations were not associated with reactivity in the various groups. In the adjusted assay, reactivity to brain antigens, as measured by PCA factor 1 score, was positively correlated with TNFα concentration. This correlation was significant for children over the age of one year (R_Spearman_ = +0.41, p = 0.02) (figure 7a). Factor 1 score was particularly high in the CM patients with the highest plasma TNFα concentrations (>100 pg/ml). The intensity of the unadjusted (but not of the adjusted) reactivity with band-0 was also correlated with TNFα concentrations (in all children: R_Spearman_ = +0.35, p = 0.008; children aged over 1 year: R_Spearman_ = +0.54, p = 0.002) (Figure 7b). Reactivity with band-0 was significantly stronger for the CM patients with the highest plasma TNFα concentrations (>100 pg/ml) than for SNCM and UM patients (p = 0.0006 and p = 0.003, respectively) (figure 7c).

## Discussion

In humans, detailed analyses of the events leading to CM and mortality from CM are restricted by ethical and practical considerations. First, sample collection is limited to peripheral blood, and post-mortem sampling is often unacceptable on cultural and religious grounds. Second, only a small proportion (ca. 1%) of the patients admitted with *P. falciparum* infection actually develops CM. Thus, it is difficult to recruit cohorts with sufficient numbers of patients meeting the CM case definition. Finally, the immuno-parasitological history of patients recruited in zones of moderate to high endemicity is generally difficult to establish, and thus the CM patients investigated might not be strictly homogeneous. Nonetheless, comparison of different cohorts with defined disease phenotypes has proven to be valuable in identifying factors specifically related to a particular pathology.

In contrast to previous studies analysing the immunoglobulin reactivity against one single antigen during malaria, in the current report we used a more global approach to analyze the response to *P. falciparum* infection without apriori forming a hypothesis about the nature of the response. In this perspective, the PANAMA-blot method coupled to computer-assisted analysis was used to investigate the global repertoire of auto-antibodies reacting to brain proteins, the organ central to CM pathology. Using this strategy associating large scale antigen-antibody reactivities and multivariate statistical analyses, we assessed the relationship between self-reactive antibodies and CM pathogenesis.

Our data showed high self-reactivity levels of IgG to brain antigens in the serum of *P. falciparum*-infected patients but not in UI or in EUIC controls. The size of the repertoire, in terms of the brain antigen diversity recognized by circulating IgG, increased significantly with disease severity. In addition, self-reactive serum IgG from 90% of CM patients displayed strong reactivity with the brain proteins found in band-0 of the Panama Blot. All the proteins in this region were demonstrated by MALDI-TOF analysis to be derived from the non-erythroid spectrin α and β chains, the α chain being more abundant. Non-erythroid spectrin is a major component of the cortical cytoskeleton of most eukaryotic cells. It forms heterodimers composed of α and β subunits [29]. The α chain of the non-erythroid spectrin (α-II spectrin) is a candidate autoantigen in primary Sjögren's syndrome [30]. This protein is cleaved during apoptosis [31], [32], and cleavage is effected following activation of a neutral calcium activated protease, calpain [32]. Interestingly, it has been recently shown that neuronal calpain is activated in *P. berghei* ANKA infected mice developing CM [33]. In addition, it is also known that inappropriate upregulation of calpain could also contribute to increased neurodegeneration in autoimmune encephalomyelitis and induced apoptosis of neuronal cells following cerebral hypoxia and ischemia [34]–[36].

We also observed that serum TNFα concentrations were correlated with brain antigen self-reactive antibodies (band-0 proteins). These concentrations were clearly higher in patients presenting severe malaria than in those admitted with uncomplicated malaria. Furthermore, the CM patients with the highest TNFα plasma concentrations (>100 pg/ml) also presented the highest levels of anti-brain reactivity. TNFα has been associated with pathogenic processes leading to CM [17]. Fever and other clinical and biological manifestations of malaria such as hypoglycemia, anemia and the adhesion of mature blood stage parasites to the brain vascular endothelium that probably decreases cerebral blood flow, leading to brain damage and coma, have been directly related to the secretion of this cytokine [18], [37]. In our study, 62.5% (5/8) of patients who died from cerebral malaria presented simultaneously high TNFα concentrations and high anti-brain reactivity levels. Thus, our data support the notion that the antibody-mediated self-reactive pathway is associated with the inflammatory process linked to CM development and would suggest that both high TNFα concentrations and high self-reactive response to brain α spectrin could be co-factors implicated in the disease severity.

The presence of self-reactive antibodies against non-cerebral antigens associated to malaria severity had been reported previously [6], [9], [11]–[13]. However, to our knowledge, the present study is the first where the whole spectrum of brain antigens, rather than a specific candidate protein, were analysed with respect to IgG autoantibodies present in the sera of a panel of patient groups representative of distinct clinical malaria phenotypes.

The function and origin of autoantibodies targeting brain protein observed in sera of CM patients is yet to be determined. In addition to the autoantibodies to α−ΙΙ spectrin, others reacting with CNS voltage-gated calcium channels were also found to be associated with an increased risk of developing severe disease in Kenyan children [3]. The production of autoantibodies to brain proteins could be considered as a signature of clinical severity in malaria.

Self-reactive antibodies to brain produced during *P. falciparum* malaria may have distinct origins including polyclonal B-cell activation or the activation of specific B-cell clones. Parasite antigens and/or parasite-derived mitogens can lead to the non-specific polyclonal activation of B lymphocytes [5], [38], and in particular to stimulation of the physiological B-cell repertoire, which has a natural capacity to react to various self- and/or environmental antigens [39]. This natural self-reactive antibody repertoire is acquired in early life and is much conserved [39]–[41]. However, if the self-reactive anti-brain protein antibodies detected in our patients were essentially due to a non-specific polyclonal B-cell activation, we would not expect any significant difference in the proportion of self-reactive antibodies or in the frequency of individuals producing these antibodies, between the different symptomatic *P. falciparum* malaria patient cohorts. It is noteworthy that our results showed such differences, thus suggesting that the induction of a specific set of brain self-reactive IgG molecules was due to the activation of a selected repertoire. Several observations are consistent with this: 1) a single band was associated with strong anti-brain reactivity in CM patients, particularly those with low IgG concentrations, 2) high anti-brain reactivity was essentially observed in the adjusted assay, in which the effects of both age and IgG concentration were reduced, and 3) IgG concentrations were not correlated with anti-brain reactivity or parasitemia. Nonetheless, the two pathways of B-cell activation, mitogenic and antigen-specific, are not mutually exclusive in the malaria infection.

The hypothesis of a role played by autoantibodies to brain proteins in CM pathophysiology is in agreement with previous observations made for numerous autoimmune diseases involving the brain [42]–[46]. For example, a role for self-reactive antibodies has been implicated in the brain damage and severe pathology observed in experimental autoimmune encephalomyelitis (EAE), a model for multiple sclerosis [46]. Moreover, it has been shown that in Rasmussen's encephalitis, a childhood autoimmune disease, the access of IgG to glutamate receptor (GluR_3_) B-cell epitopes in the central nervous system triggers complement-mediated neuronal damage and contributes to the pathogenesis [45]. In neuropsychiatric systemic lupus erythematous, antibodies to microtubule-associated protein 2 (MAP-2) and to cardiolipin were also associated with the pathology [43], [44]. Interestingly, the serum of these patients contains auto-antibodies to the NMDA receptors [42], and these elicit cognitive impairment in mice that received lipopolysaccharide to compromise the blood brain barrier integrity [42]. Generally speaking, a role of viral or bacterial infections has been suggested in the development of autoimmune diseases [47]–[50]. For example, an autoimmune process associated with brain damage and dementia has been observed in HIV infected patients [51].

An organized core autoimmune repertoire within the immune system has been termed the immunological homunculus [52]. According to this concept, high levels of α−ΙΙ spectrin autoantibodies would not be caused by the direct antigenic stimulation of anti-brain α−ΙΙ spectrin lymphocytes clones, but rather the disease manifestation (CM) would be a consequence of a *P. falciparum*-induced defect or weakening of the natural regulation of homunculus autoreactivity [53], [54]. Alternatively, the inflammation associated with the sites of parasitized and non-parasitized red blood cells sequestration and brain ischemia could lead to neuronal damage and exposure of self-antigens which in turn will elicit a self-reactive antibody response, thus amplifying brain damage [18]–[22]. Observations of this process in various pathological conditions such as brain contusion [55] and hypoxia [56] support this hypothesis. Therefore, the presence of self-reactive antibodies to α-II spectrin might be consequent to parasite exposure, and their levels might simply parallel the degree of neuronal damage. In this context, these antibodies would not be implicated in the pathogenic process, but rather behave as a marker of severity. In both hypothesis, and by analogy to tissue-specific autoimmune diseases, the presence of antibodies recognizing self antigens may amplify any neuronal damage resulting from the inflammation initiated by parasite sequestration in the brain [57]. In this case, these antibodies would exacerbate the pathogenic process. Clearly, the role of self-reactive antibodies in the pathogenesis of CM and other severe manifestations of malaria should be investigated further in different cohorts from endemic regions. Furthermore, it will be of interest to determine whether cytophilic antibodies to α-II spectrin play a role in neuropathogenesis since the presence of cytophilic antibodies in the serum of rats immunized with rat myelin basic protein (MBP) has been reported in EAE [58].

Another mechanism which may explain the genesis of an autoimmune B cell repertoire to brain antigens linked to the neuropathology of malaria could be Fas-derived apoptosis during the course of the fatal disease, as it has been suggested for experimental cerebral malaria in macaques and mice [59], [60]. This notion is supported by the observation that CM incidence was reduced in Fas/Fas-L-deficient mice [61]. In this context, a significant increase of plasma levels of sFas-L with disease severity was noted previously in malaria patients [62], as well as in a study we undertook in the same cohorts of patients (unpublished results). Neuronal cell death by apoptosis during CM could then be a potential source of autoantigens that may lead to stimulation of the autoimmune responses [63].

In conclusion, the observation that children exposed to *P. falciparum* malaria have antibodies to brain antigen in their plasma, may be the consequence of a cumulation number of sub-clinical brain damage resulting from previous episodes of severe non-cerebral malaria. Irreversible damage due to acute and chronic malaria episodes has been recorded in experimental infections of mice with *Plasmodium* [64]. Repeated malaria episodes would lead to higher levels of anti-brain antibodies of increasing complexity in terms of antigen repertoire specificity. The risk of developing cerebral malaria would then increase with the presence and level of these antibodies. An initial parasite-mediated lesion in the brain causing neuronal damage and leakage in the blood brain barrier will allow serum autoantibodies to bind to their cognate brain antigens leading to complement activation and further inflammation, oedema, and brain damage. Clearly, it would be interesting to determine the prevalence and levels of anti-α-II spectrin autoantibodies in endemic residents. If two cohorts distinguished by significantly different levels of these antibodies could be constituted, then it will be possible to conduct a prospective study that could allow to determine whether high anti-αII spectrin autoantibody levels predispose an individual to develop CM.
